# Being Anxious, Thinking Positively: The Effect of Emotional Context on Respiratory Sensory Gating

**DOI:** 10.3389/fphys.2016.00019

**Published:** 2016-02-16

**Authors:** Pei-Ying S. Chan, Chia-Hsiung Cheng, Ya-Jhih Jhu, Chia-Ling Chen, Andreas von Leupoldt

**Affiliations:** ^1^Department of Occupational Therapy, College of Medicine, and Healthy Ageing Research Center, Chang Gung UniversityTaoyuan, Taiwan; ^2^Department of Psychiatry, Chang Gung Memorial Hospital at LinkouTaoyuan, Taiwan; ^3^Division of Psychiatry, Taipei Veterans General Hospital at Yuan ShanYilan County, Taiwan; ^4^Department of Physical Medicine and Rehabilitation, Chang Gung Memorial hospital at LinkouTaoyuan, Taiwan; ^5^Graduate Institute of Early Intervention, College of Medicine, Chang Gung UniversityTaoyuan, Taiwan; ^6^Health Psychology, University of LeuvenLeuven, Belgium

**Keywords:** respiratory sensory gating, positive emotional context, respiratory sensation, respiratory-related evoked potential (RREP), anxiety

## Abstract

Respiratory sensory gating function has been found decreased by induced negative emotion in healthy adults. The increased ratio of the respiratory-related evoked potential (RREP) N1 peak amplitude for the second occlusion (S2) vs. the first occlusion (S1), S2/S1, is indicative of such decreased respiratory sensory gating. In this study, we tested the hypothesis that a positive emotional context would enhance respiratory sensory gating function in healthy individuals. In addition, we tested the modulating role of individual anxiety levels. We compared respiratory sensory gating in 40 healthy individuals by the paired inspiratory occlusion paradigm in a positive and neutral emotional context induced by emotional picture viewing. The results showed that the group averaged RREP N1 peak amplitudes S2/S1 ratios were significantly smaller in the positive compared to neutral context (0.49 vs. 0.64; *p* < 0.01). Further, analysis showed that the ratio decrease was due to a reduced response to the S2 and an enhanced response to S1 in the positive emotional context (*p* < 0.05). The subgroup analyses showed that in the positive emotional context, both individuals with low-moderate anxiety levels and those with no anxiety demonstrated a significant decrease of their S2/S1 ratio, but only those with low-moderate anxiety levels showed reduced S2 amplitudes compared to the neutral context (*p* < 0.01). In conclusion, our results suggest that a positive emotional context is related to better brain inhibitory mechanisms by filtering out repetitive respiratory stimuli in healthy individuals, especially in the presence of low-moderate anxiety levels. Further, investigation on how positive emotional contexts might contribute to improved respiratory sensory gating ability in clinical populations is necessary.

## Introduction

The perception of respiratory sensations is of essential importance for managing symptoms in respiratory diseases such as asthma and chronic obstructive pulmonary disease (COPD), but it is also relevant in anxiety disorders (Tiller et al., 1987; Rietveld, 1998; von Leupoldt and Dahme, 2007; Janssens et al., 2009; Paulus and Stein, 2010). However, the relationship between the subjective perception of respiratory sensations and objective respiratory impairment is often weak (Brand et al., 1992; Bijl-Hofland et al., 1999; Wamboldt et al., 2000; Boulet and Turcotte, 2007). For example, some patients with relatively normal pulmonary function might report severe subjective dyspnea, whereas other patients with low pulmonary function might report only very mild symptoms (Janssens et al., 2009). Therefore, several impacting mechanisms unrelated to objectively-measured pulmonary function have been suggested over the past years (Janssens et al., 2009; Hayen et al., 2013; Laviolette et al., 2014). In this regard, especially the relationship between emotional factors and subjective perception of dyspnea has been studied over the past decade (von Leupoldt et al., 2010a).

These studies have shown that negative emotional states as well as personality traits such as anxiety are related to increased perception of respiratory sensations, whereas positive emotional states were related to reduced-perception of respiratory sensations. However, the number of studies that, in addition to subjective reports, objectively measured emotional effects on respiratory perception (for example by using electrophysiological recordings) has only recently grown (for review, see von Leupoldt et al., 2013). For example, the method of respiratory-related evoked potentials (RREP) in the electroencephalogram (EEG) has been used to study cortical neural processing elicited by respiratory mechanical stimuli in both humans and animals. These cortical dipole recordings of cerebral neuronal activations during the processing of information from respiratory muscle afferents are identified with the short- and long-latency peaks of Nf, P1, N1, P2, and P3 (Chan and Davenport, 2010). The short-latency exogenous (i.e., being affected majorly by the external factors such as stimulus intensity and airway resistance) peaks of Nf and P1 were found related to respiratory stimulus discrimination (Knafelc and Davenport, 1999; Davenport et al., 2012), while the long-latency endogenous (i.e., being affected majorly by the internal factors such as focused attention and emotion) peaks of P2 and P3 were found related to the affective dimension of respiratory perception (von Leupoldt et al., 2010b; Chan et al., 2014). The N1 peak is thought to reflect both exogenous and endogenous aspects as it was found affected by manipulating physiological factors (Chou and Davenport, 2007; Davenport et al., 2007), but also by manipulating psychological factors (Harver et al., 1995; Webster and Colrain, 2000).

A few recent studies using the RREP investigated the relationship between emotionality or emotional context and respiratory perception (von Leupoldt et al., 2010b, 2011; Chan et al., 2014). For example, using the single inspiratory occlusion RREP paradigm, a negative emotional context (e.g., viewing negative emotional pictures) was found to result in limited usage of attentional resources for respiratory sensation as evidenced by decreased RREP P2 and P3 amplitudes (von Leupoldt et al., 2010b, 2011). However, use of the single-occlusion method could not adequately explain over-perception of respiratory sensations. Therefore, the paired occlusion paradigm emerged to test sensory gating or sensory flooding phenomena in respiration (Chan and Davenport, 2008).

Respiratory sensory gating, similar to other types of neural gating with exteroceptive stimuli such as sound and touch (Adler et al., 1982; Arnfred et al., 2001), has been used to investigate the central neural mechanism of filtering repetitive respiratory stimuli within a short time window (Chan and Davenport, 2010). The paired-obstruction stimulation paradigm is used to examine respiratory sensory gating by eliciting paired RREP waveforms where the second stimulus (S2) results in a smaller N1 peak amplitude compared to the first stimulus (S1) in healthy individuals (Chan and Davenport, 2008). A smaller ratio for the RREP N1 responses to the S2 vs. S1 (as represented by S2/S1 ratio) is indicative of a better respiratory sensory gating function (i.e., filtering out more repetitive sensory information). A few recent studies have tested the effect of negative emotional context or personality traits on respiratory sensory gating and found that negative emotional stimulation decreased the respiratory gating function (Chenivesse et al., 2014), and that individuals with higher anxiety levels and patients with Generalized Anxiety Disorder (GAD) compared to healthy control subjects demonstrated a reduced respiratory gating function by an increased RREP N1 amplitude S2/S1 ratio along with enhanced S2 amplitudes (Chan et al., 2012, 2015). However, respective effects of positive emotional contexts on respiratory sensory gating are widely unknown.

Therefore, the present study tested the effect of a positive emotional context induced by emotional picture viewing on respiratory sensory gating in healthy individuals. In line with previous behavioral findings (Rietveld et al., 2000; von Leupoldt et al., 2006a,b; Janssens et al., 2009), we hypothesized that a positive emotional context would be associated with better respiratory sensory gating function. In addition, we tested how individuals' anxiety levels would influence this association.

## Materials and methods

### Subjects

A group of 40 non-smoking healthy adults without self-reported respiratory, cardiovascular, or neurological diseases were recruited through verbal and online bulletin board advertisements. After the arrival in the laboratory, the subjects were provided with a detailed informed consent form with explanations regarding the nature of the study. After the informed consent was obtained, the subjects performed a pulmonary function test (PFT) with a standard spirometry device (Cardinal Health Inc., Dublin, OH, USA) for the determination of the Forced Expiratory Volume in 1 s (FEV1). The PFT was conducted based on the guidelines of the American Thoracic Society and European Respiratory Society (Miller et al., 2005). All subjects passed the minimum requirement of FEV1 > 70% of predicted norm values in order to be included. The subjects were then administered with the Chinese version of the Beck Anxiety Inventory (BAI) (Beck and Steer, 1990). The BAI is a 4-point rating scale with 21 questions. For each question, the subjects report the degree to which they are bothered by their emotional experiences ranging from 0 (not at all) to 3 (could barely stand it). The study protocol was approved by the Institutional Review Board of the Chang Gung Medical Foundation.

### Experimental procedure

#### Respiratory apparatus

For details of the respiratory apparatus setup, refer to Chan et al. (2012). In the present study, the participant was instructed to sit comfortably in an armchair while breathing through a face mask connecting to a two-way non-rebreathing valve (Hans Rudolph Inc., Kansas City, USA). The face mask was attached to the subject and the non-rebreathing valve was suspended in order to minimize facial movements and to maximize the subject's comfort. The non-rebreathing valve was connected to a customized occlusion valve (Hans Rudolph Inc., Kansas City, USA), controlled by a solenoid, which connected to a pressure tank through reinforced tubing (Chan et al., 2015). Paired inspiratory occlusions were elicited manually by the experimenter via a trigger box in the adjacent room. When the trigger was activated, the occlusion valve produced a transient closure of the inspiratory port of the apparatus. The change in mouth pressure was monitored via a differential pressure transducer connected to a pneumotachograph amplifier (1110 series, Hans Rudolph) and a PowerLab signal recording unit (ADInstruments Inc., Bella Vista, Australia).

#### RREP paired occlusion paradigm

**Figure 1:**
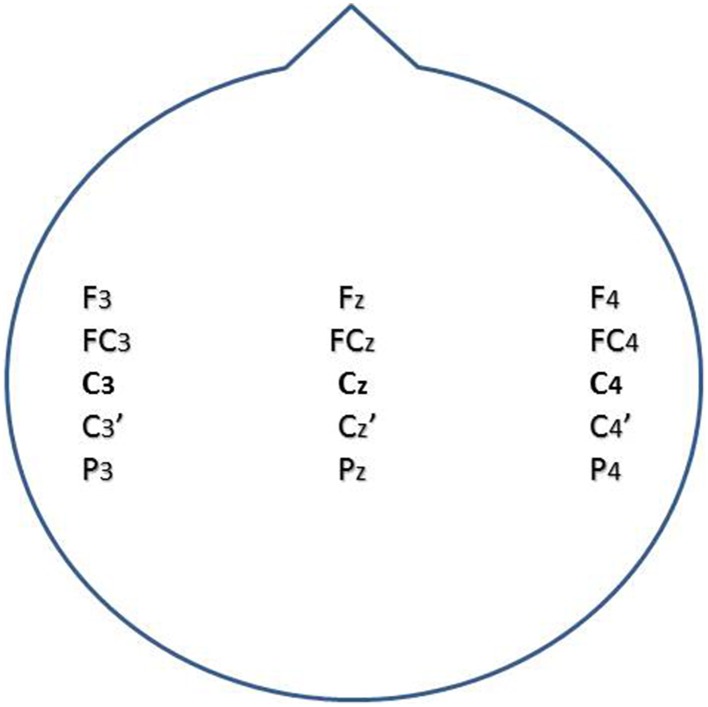
**A schematic representation for the position of electrodes for EEG recordings**.

An electrode cap based on the International 10–20 system was positioned on the subject's head by the experimenter. Figure 1 shows a schematic representation of the electrode sites of the cap. For a detailed review on the EEG setup, please refer to Chan and Davenport's (2008) review. Before the recording, the subjects were familiarized with the sensations of the paired inspiratory occlusions. Two inspiratory occlusions of 150 ms each, with a 500 ms inter-stimulus-interval, were provided randomly every 2–4 breaths. The onset of occlusion was identified as the start of mouth pressure change (Labchart V7, ADInstruments Inc., Bella Vista, Australia). Approximately 100 paired occlusions were provided during each of the two trials. The trigger box was sending parallel markers to the EEG recording software (Neuroscan 4.5, Compumedics Neuroscan Inc., Charlotte, NC, USA). The continuous EEG was sampled at 1 kHz with a 40-channel EEG system (NuAmps, Compumedics Neuroscan Inc., Charlotte, NC, USA), bandpass filtered from DC to 50 Hz and referenced to the bilateral mastoids. Individual electrode impedance was set below 5 kΩ. After each trial, the subjects were rating their subjective feeling of breathlessness with a visual analog scale (VAS) (0 = not at all breathlessness, and 100 = maximal level of breathlessness).

#### The emotional picture series

A total of two trials with a 5-min rest in between were performed consecutively in an experiment. In order to induce an emotionally positive and neutral context, one trial was performed while in parallel positive emotional pictures were presented, whereas the other trial was performed while presenting emotional neutral pictures. The order of the trials was randomized across subjects. The emotional pictures were pre-selected from the International Affective Picture System (IAPS) (Lang et al., 2008). The IAPS is a widely used and standardized instrument for the induction of different emotional states. Both picture series consisted of 120 pictures which were presented randomly within the trial. Each picture was presented for 6 s on a monitor placed in front of the subjects. The pictures series were presented with the software E-prime (Psychology Software Tools, Pittsburgh, PA). After each trial, the subjects rated their perceived level of emotional valence and arousal using the 9-point Likert scale of the Self-Assessment Manikin (SAM) (Bradley and Lang, 1994). The scores for the valence and arousal level range from 1 (unpleasant/relaxed state) to 9 (pleasant/aroused state).

### Data analysis

For the paired RREP, the EEG epoch was defined and extracted from 200 ms before to 500 ms after the onset of the inspiratory occlusions separately for the S1 and S2 RREP. The first 200 ms served as baseline for the signal. The EEG signals were processed with ocular movement correction using a built-in algorithm of the BrainVision Analyzer 2 software (Brain Products GmbH, Gilching, Germany) and bandpass filtered between 1 and 30 Hz (12 dB/octave roll-off). The artifacts were defined as more than 100 and 60 μV, baseline to peak, for the four eye electrodes and all the other electrodes, respectively. After the identified artifacts were extracted from the electrodes, the corresponding epochs were then averaged for S1 and S2.

According to the past studies in RREP source localizations, the N1 peak is observed maximal at the vertex (Cz electrode) (Logie et al., 1998) and at the bilateral sensorimotor cortices (C3 and C4 electrodes) (von Leupoldt et al., 2010c). Therefore, the RREP N1 peak was identified as the second negative peak maximal over the central region between 85 and 135 ms after the occlusion (electrodes around C3, Cz and C4) in the present study. Peak amplitudes for the RREP N1 S1 and S2, and the S2/S1 ratios were calculated. One-way repeated measure analyses of variance (RMANOVA) were performed for the RREP N1 peak amplitude S2/S1 ratios as well as the subjective ratings on the VAS and SAM scales to examine the effect of emotional contexts by using SPSS (SPSS Inc., Chicago, IL, USA). Subsequent analyses were then performed to test whether effects were due to changes in subjects' N1 S1 and/or N1 S2 amplitudes. Finally, we conducted subgroup analyses where we divided the 40 subjects, based on a median split of their BAI summary scores, into two groups (20 low-moderately anxious vs. 20 non-anxious subjects). More specifically, we examined the differences regarding the S2/S1 ratios, N1 S1 and N1 S2 amplitudes as well as the ratings on VAS and SAM between the subgroups with independent *t*-tests. The critical *p*-value was set at 0.05.

## Results

Forty-three healthy non-smoking subjects participated in this study. None of the participants had a history of substance or alcohol abuse. Three individuals' data were excluded because the quality of the signals was severely affected by artifacts during recording, which left the study with 40 subjects (28 females and 12 males) for final analyses. The demographic data and the PFT results of the 40 subjects are shown in Table 1. Table 1 also lists the characteristics of the two subgroups divided by the median score of the BAI ratings.

**Table 1:** **Basic characteristics of study subjects including the subgroups divided by BAI scores (Group Mean ± SD)**.

**Variables**	**All subjects**	**LMA**	**NA**
*N*	40	20	20
Age (y/o)	23.8 ± 4.2	24.8 ± 5.38	22.9 ± 1.93
Gender (female/male)	28/12	15/5	13/7
FEV1 (L)	3.08 ± 0.62	3.04 ± 0.63	3.11 ± 0.61
FEV1 of predicted value (%)	81.13 ± 7	81.8 ± 9.06	80.45 ± 6.22
BAI	8.9 ± 9.48	15.25 ± 9.77	2.55 ± 1.88^*^

*The asterisk indicates a statistical difference between the two subgroups (p < 0.05)*.

### Affective ratings

The subjective ratings of emotional valence were significantly higher in the pleasant compared to neutral context (7.45 ± 1.09 and 4.95 ± 1.09, respectively, *p* < 0.001). The ratings for the emotional arousal level did not differ significantly between the two contexts (4.03 ± 2 for the pleasant and 3.65 ± 2.33 for the neutral context). In the subgroup analysis, the arousal ratings were similar between the two subgroups. In the positive emotional context, the higher anxious subjects rated the level of pleasure (valence) higher than the lower anxious subjects (7.8 ± 1.08 and 7.1 ± 0.99, respectively, *p* < 0.05).

### Perceived breathlessness ratings

The subjective ratings of breathlessness were higher in the neutral than in the positive emotional context (42.86 ± 24.04 and 35.2 ± 24.7, respectively, *p* < 0.05). In the subgroup analysis, no differences were observed between the two subgroups for both contexts.

### RREP results

**Figure 2:**
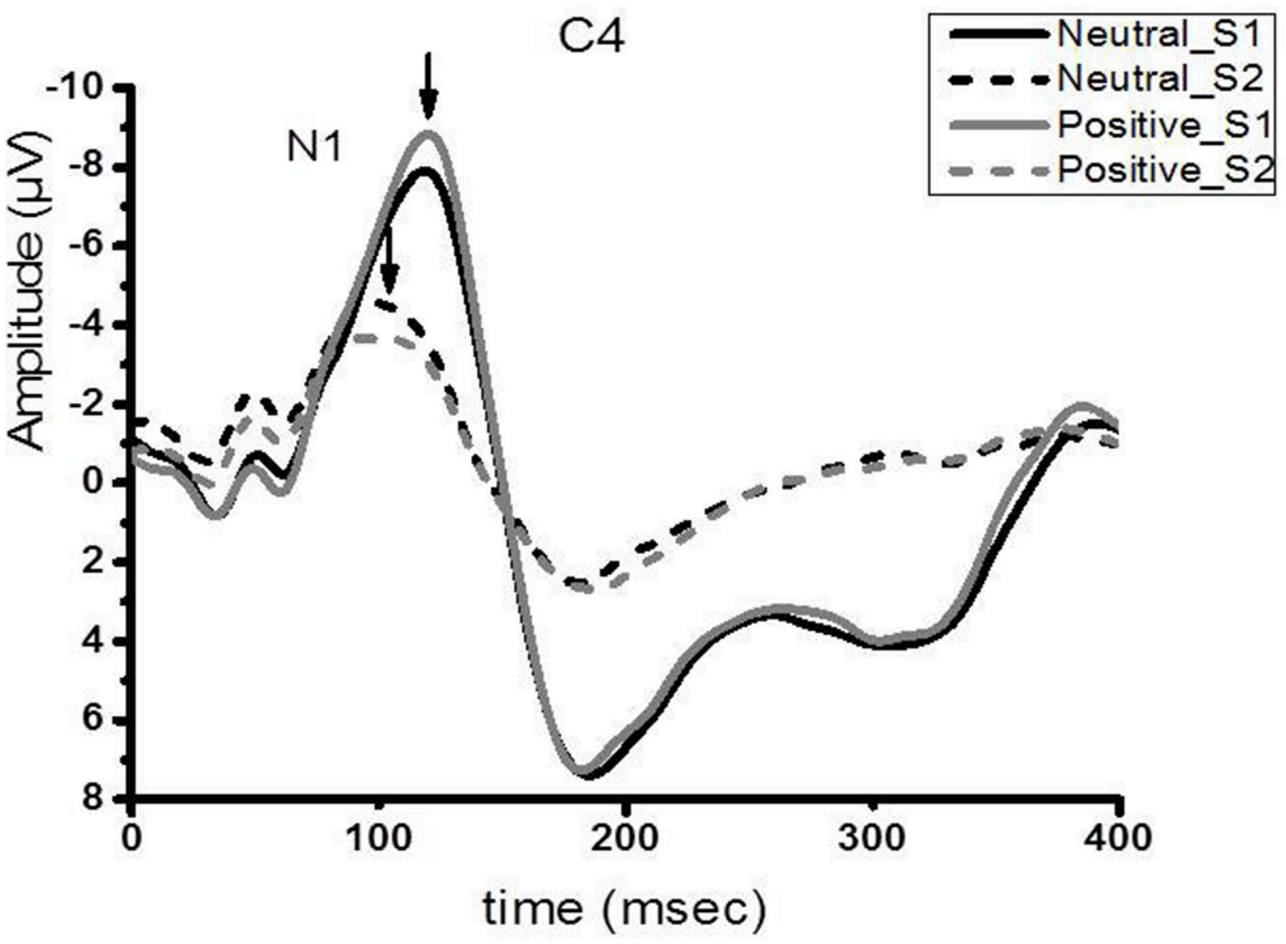
**Group averaged (***N*** = 40) waveform from the C4 electrode**. The black solid and dotted lines represent the averaged S1 and S2 waveforms in the neutral context, respectively. The gray solid and dotted lines represent the averaged S2 waveforms, respectively, in the positive context.

**Figure 3:**
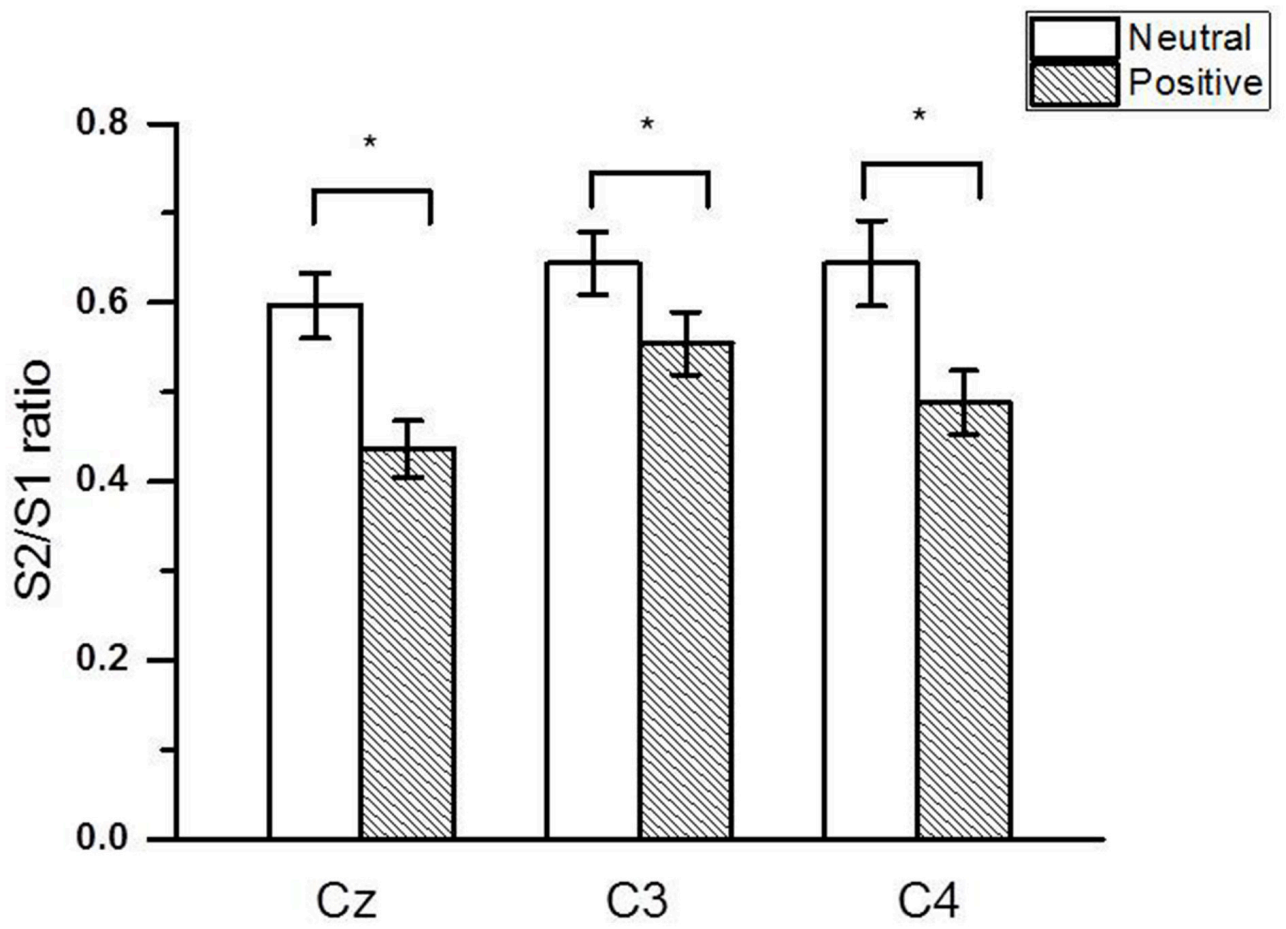
**The group averaged (± SE) (***N*** = 40) RREP N1 peak gating ratios at the Cz, C3, and C4 electrodes**. The asterisk indicates a significant difference between the two emotional contexts (*p* < 0.05).

Figure 2 shows the group-averaged S1 and S2 RREP waveforms in the two different emotional contexts. The One-way RMANOVA revealed a significantly smaller N1 amplitude S2/S1 ratio in the positive compared to the neutral emotional context (*p* < 0.05). Further, analyses showed increased N1 S1 and decreased N1 S2 amplitudes in the pleasant context compared to the neutral context (*p*'s < 0.05). Table 2 shows the calculated N1 S1 and N1 S2 amplitudes for the Cz, C3, and C4 electrodes as well as the subjective ratings in the two emotional contexts. Figure 3 shows a bar graph of the N1 amplitude S2/S1 ratios for the Cz, C3, and C4 electrodes in both emotional contexts.

**Table 2:** **Group averaged (±SD) (***N*** = 40) RREP N1 peak S2/S1 ratios at the central electrode sites as well as the subjective ratings of breathlessness, valence, and arousal in the two emotional contexts**.

**Parameter**	**Electrode**	**Neutral context**	**Positive context**
S1	Cz	−9.9±4.22 μV	−10.47±4.91 μV
	C3	−8.96±3.64 μV	−10.19±4.76 μV^*^
	C4	−8.78±3.87 μV	−9.89±4.63 μV^*^
S2	Cz	−5.51±2.61 μV	−4.32±2.13 μV^*^
	C3	−5.29±1.69 μV	−5.09±2.17 μV
	C4	−5.05±2.03 μV	−4.32±1.91 μV^*^
Breathlessness		42.86±24.04	35.2±24.7^*^
Valence		4.95±1.09	7.45±1.09^*^
Arousal		3.65±2.33	4.03±2.01

*The asterisk indicates a statistical difference between the neutral and positive emotional context (p < 0.05)*.

**Figure 4:**
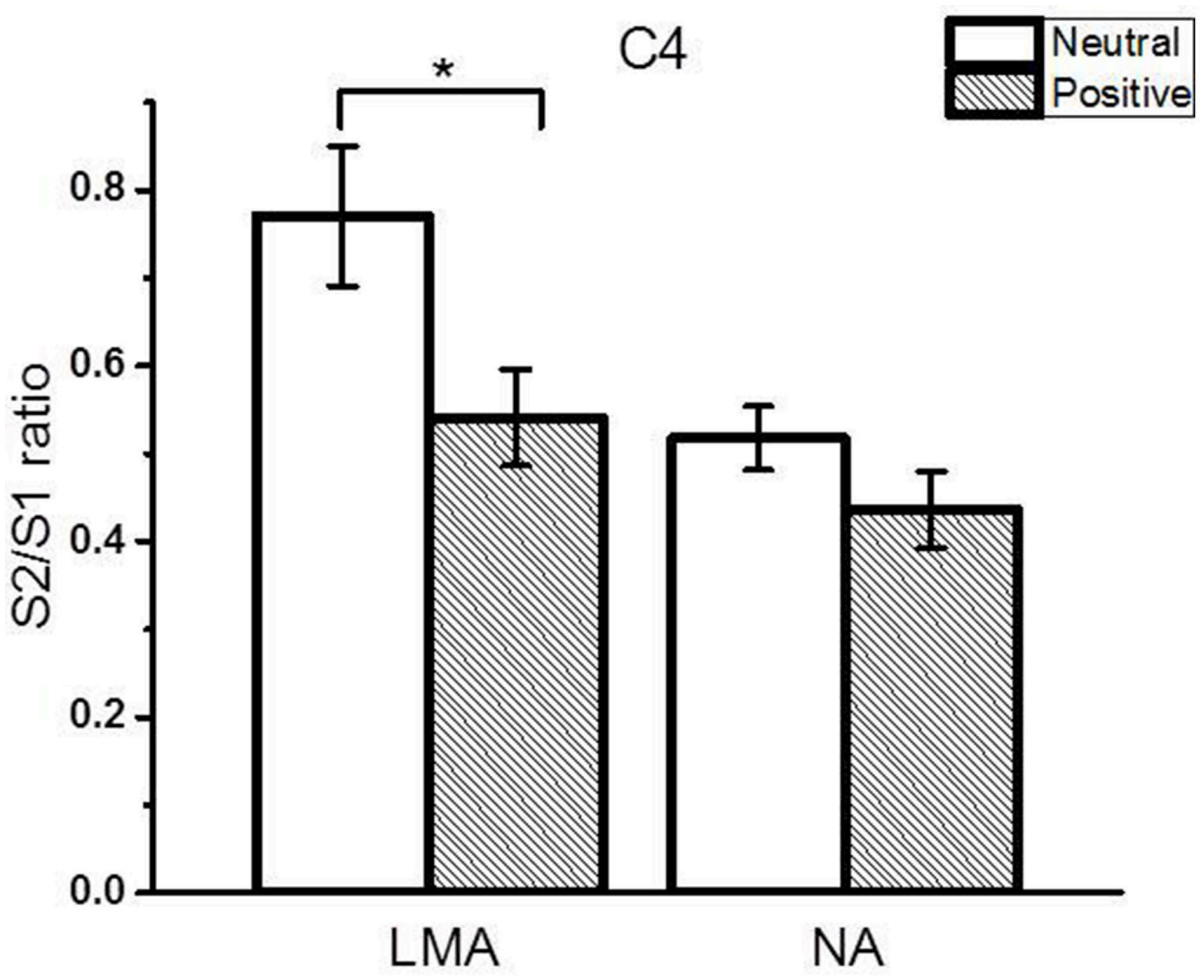
**The RREP N1 peak gating ratios (average ± SE) at the C4 electrode in the LMA (***N*** = 20) and NA (***N*** = 20) subjects in the subgroup analysis**. The asterisk indicates a statistical difference between the two emotional contexts (*p* < 0.05).

The subgroup analyses revealed that both the low-moderately anxious (LMA) and non-anxious (NA) subjects showed significantly smaller N1 amplitude S2/S1 ratios in the positive relative to the neutral context (LMA: Cz and C4, *p* < 0.05; NA: Cz and C3, *p* < 0.05). Figure 4 shows the N1 peak amplitude S2/S1 ratios of the C4 electrode in the LMA and NA subgroups, respectively, in the two emotional contexts. Further, comparisons showed that the LMA subjects demonstrated significantly reduced S2 amplitudes in the positive compared to the neutral context (Cz and C4, *p* < 0.01, see Table 3A), which was not observed in the NA subjects. Instead, the NA subjects showed a trend of smaller S1 amplitude in the neutral relative to the positive context (C3 and C4, *p* < 0.1; see Table 3B).

**Table 3:** **Group averaged (±SD) RREP N1 peak amplitudes at the central electrode sites and subjective ratings for the (A) LMA (***N*** = 20) and (B) NA (***N*** = 20) subgroups in the two emotional contexts**.

**Parameter**	**Electrode**	**Neutral context**	**Positive context**
***(A) THE LMA SUBGROUP***			
S1	Cz	−8.82±4.96 μV	−8.66±4.96 μV
	C3	−8±4.41μV	−9.16±5.73 μV
	C4	−7.8±4.86 μV	−8.33±4.78 μV
S2	Cz	−5.25±2.46 μV	−3.82±2.17 μV^*^
	C3	−5.11±1.97 μV	−4.92±2.41 μV
	C4	−5.1±2.3 μV	−3.89±1.65 μV^*^
Breathlessness		48.65±20.02	39.85±22.12^*^
Valence		4.75±1.13	7.8±1.08^*^
Arousal		4±2.12	4.2±1.96
***(B) THE NA SUBGROUP***			
S1	Cz	−10.91±2.8 μV	−12.22±4.16 μV
	C3	−9.91±2.27 μV	−11.52±3.34 μV
	C4	−9.71±2.12 μV	−11.46±3.88 μV
S2	Cz	−5.76±2.7 μV	−4.79±5.91 μV
	C3	−5.47±1.41 μV	−5.25±1.88 μV
	C4	−5.01±1.71 μV	−4.75±2.04 μV
Breathlessness		37.08±26.24	30.55±26.23^*^
Valence		5.15±1.01	7.1±0.99^*^
Arousal		3.3±2.47	3.85±2.03

*The asterisk indicates a statistical difference between the two contexts (p < 0.05)*.

## Discussion

The major results of the present study supported our hypothesis that a positive emotional context enhances respiratory sensory gating functions in response to repetitive respiratory stimuli. Our results of a decreased RREP N1 peak S2/S1 ratio as a function of enhanced S1 response and reduced S2 response in the positive relative to the neutral context provide additional information to the past RREP gating studies examining the effects of emotion (Chan and Davenport, 2010; Chan et al., 2012, 2015; Chenivesse et al., 2014). For example, Chan and Davenport (2010) found that an anxious state induced by nicotine withdrawal was associated with decreased respiratory gating function as indicated by an increased N1 S2 response. Chenivesse et al. (2014) examined the impact of negative emotional context on respiratory gating and found reduced gating function compared to a neutral emotional context with decreased S1 amplitudes in the negative emotional context. Other studies have similarly found that transient negative emotional states as well as personality traits such as generalized anxiety disorder are related to altered respiratory sensory gating by either increased S2 responses (Chan et al., 2012) or decreased S1 responses (Chan et al., 2015). Taken together the above findings, it seems that a transient anxious state—as examined in healthy samples—has influences on respiratory neural responses to both the first, incoming stimulus (reduced S1 signal) and the repetitive stimulus (enhanced S2 signal), whereas in clinical patients with generalized anxiety disorder, only a compromised response to S1 was observed. However, whether this discrepancy between healthy subjects and clinical populations reflects an underlying difference in malfunctioning filtering circuits or a limited usage of neural attentional resources needs to be elucidated in future studies.

In addition, our subgroup analysis revealed that compared to a neutral context, only the LMA subgroup demonstrated a significantly reduced S2 response in a positive emotional context, which was not observed in the NA subgroup. Although there is no direct evidence linking the current finding with fewer symptom reports in anxious individuals in the positive emotional context, the current finding of “less sensory flooding in the cortex” is parallel to some previous behavioral evidence. Positive emotions including humor and transient positive mood states were associated with less respiratory symptom report in healthy controls as well as patient populations (Bogaerts et al., 2005; von Leupoldt et al., 2006a, 2010a; Rietveld and van Beest, 2007). For instance, children with asthma reported lower resistive-load induced breathlessness in a positive emotional context relative to a neutral context induced by watching emotional film clips (von Leupoldt et al., 2006a). Similarly, COPD patients who were performing cycle ergometer exercise reported lower level of breathlessness when viewing positive compared to negative emotional pictures (von Leupoldt et al., 2010a). In addition, our result is in line with the notion that use of self-selected music during exercise in pulmonary rehabilitation programs is associated with less dyspnea reports than exercise without listening to music in COPD patients (Bauldoff et al., 2002; von Leupoldt et al., 2007), since self-selected music is usually reasoned to provide individuals with a positive emotional experience. Moreover, the present finding of decreased subjective ratings of breathlessness in response to respiratory occlusions in the positive emotional context further supports our electrophysiological results. The above evidence suggests that these findings might be related to the improved respiratory sensory gating function in positive emotional contexts.

Previous research has suggested that behavioral medicine approaches such as cognitive behavioral therapy added to pulmonary patients' treatment might not only reduce levels of negative emotions such as depression and anxiety (Coventry and Hind, 2007; Stewart and Chambless, 2009), but also levels of respiratory symptoms (Griffiths et al., 2000; White et al., 2002; Coventry and Hind, 2007; Livermore et al., 2015). Our results support these findings by demonstrating measurable objective changes in a positive emotional context on the neural processing of such respiratory sensations. With the neurophysiological evidence measured in the healthy population, our finding implicates the importance of creating an environment of positive valence as an intervention technique for relieving respiratory symptoms in patients with respiratory or psychological diseases. Given the non-clinical anxiety levels in the present sample, future research is recommended to examine electrophysiological evidence for the effects of positive emotional context on respiratory perception in clinical populations including patients with respiratory disease with and without clinically relevant anxiety.

In summary, the present study suggests that a positive emotional context is associated with a better respiratory sensory gating function evidenced by smaller N1 peak amplitude S2/S1 ratios. Moreover, this improved neural filtering of repetitive respiratory stimuli is modified by individual anxiety levels, even in healthy non-clinically anxious subjects. Future research is therefore needed to examine the effects of positive emotional contexts on respiratory gating in clinical populations.

## Author contributions

PC contributed to the conception or design of the work; or the acquisition, analysis, and interpretation of data for the work; CC and YJ contributed majorly to the design of the work, acquisition and analysis of the data whereas CC and AV contributed majorly to the design and interpretation of the data. All authors contributed to the drafting the work or revising it critically for important intellectual content, final approval of the version to be published, agreement to be accountable for all aspects of the work in ensuring that questions related to the accuracy or integrity of any part of the work are appropriately investigated and resolved.

### Conflict of interest statement

The authors declare that the research was conducted in the absence of any commercial or financial relationships that could be construed as a potential conflict of interest.
